# Assessment of malignancy and PSMA expression of uncertain bone foci in [^18^F]PSMA-1007 PET/CT for prostate cancer—a single-centre experience of PET-guided biopsies

**DOI:** 10.1007/s00259-022-05745-5

**Published:** 2022-04-28

**Authors:** Bernd Vollnberg, Ian Alberts, Vera Genitsch, Axel Rominger, Ali Afshar-Oromieh

**Affiliations:** 1 Department of Nuclear Medicine, Inselspital, Bern University Hospital, University of Bern, Freiburgstr. 18, CH-3010 Bern, Switzerland; 2 Institute of Pathology, University of Bern, Bern, Switzerland

**Keywords:** Prostate cancer, PET/CT, PSMA, Prostate-specific membrane antigen, [^18^F]-PSMA-1007, Biopsy, PET-guided

## Abstract

**Purpose:**

Uncertain focal bone uptake (UBU) with intensive radiopharmaceutical avidity are frequently observed in patients undergoing [^18^F]PSMA-1007 PET/CT for the detection of prostate cancer (PC). Such foci can pose diagnostic conundrums and risk incorrect staging. The aim of this short communication is to share the results of PET-guided biopsies of such foci.

**Methods:**

A retrospective analysis revealed 10 patients who were referred to our department for PET-guided biopsy of UBU visible in a previous [^18^F]PSMA-1007 PET/CT. [^18^F]-PSMA-1007 PET-guided biopsy was conducted for 11 PSMA-avid bone foci in these 10 patients. The biopsy materials were analysed for tissue typing, and immunohistochemistry (IHC) was performed for prostate-specific-membrane-antigen (PSMA) expression. The scans were analysed by two experienced physicians in a consensus read for clinical characteristics and radiopharmaceutical uptake of foci.

**Results:**

One out of 11 (9.1%) of the foci biopsied was confirmed as bone metastasis of PC with intense PSMA-expression, while 10/11 (90.9%) foci were revealed to be unremarkable bone tissue without evidence of PSMA expression at IHC. Amongst all bone foci assessed by biopsy, eight were visually classified as being at high risk of malignancy in the PET/CT (SUVmean 12.0 ± 8.1; SUVmax 18.8 ± 13.1), three as equivocal (SUVmean 4.6 ± 2.1; SUVmax 7.2 ± 3.0) and zero as low risk. No UBU had any CT correlate.

**Conclusions:**

This cohort biopsy revealed that a small but relevant number of UBU are true metastases. For those confirmed as benign, no PSMA expression at IHC was observed, suggesting a non-PSMA mediated cause for intensive [^18^F]PSMA-1007 uptake of which the reason remains unclear. Readers must interpret such foci with caution in order to reduce the risk of erroneous staging and subsequent treatment. PET-guided biopsy, particularly in the absence of morphological changes in the CT, can be a useful method to clarify such foci.

**Supplementary Information:**

The online version contains supplementary material available at 10.1007/s00259-022-05745-5.

## Introduction

First introduced clinically in 2011 [1], combined positron emission and computed tomography (PET/CT) using the PSMA-radioligand [^68^ Ga]Ga-PSMA-11 has established itself as the gold standard for the imaging of recurrent PC. More recently, [^18^F]-radiolabelled PSMA ligands have been introduced and have a number of potential advantages when compared to [^68^ Ga] [2]. Amongst these tracers is [^18^F]PSMA-1007, for which previous studies suggest a higher PET-positivity rate [3]. However, there are a number of reports of high rates (in up to half of all patients) of unspecific or equivocal bone foci (UBU), which cannot be clearly classified as benign or malignant [4, 5]. These can often pose diagnostic conundrums and place the patient at risk of being incorrectly staged; this could adversely impact treatment outcomes or might require further diagnostic workup [6]. Furthermore, with increasingly sensitive digital and ultra-long field-of-view PET/CT systems available, this problem may increase yet further in importance [5, 7–9].

Despite widespread implementation, including at our own centre, there is only limited evidence regarding the diagnostic accuracy of [^18^F]PSMA-1007, particularly for bone foci. The available literature seldom reports any comparative imaging data or uses a reference standard to confirm equivocal PET findings [3], and thus represents an urgently unmet need in evidence-based imaging for PC. Although follow-up data for a small cohort of patients with UBU in primary PC are reported [10], no similar data with a histopathological reference standard has been published for UBU in a biochemically recurrent setting, where histological confirmation is less readily available. PET-guided biopsies are a useful and minimally invasive method of obtaining material for biopsy, particularly where in the absence of morphological findings in the CT, conventional CT-guided biopsy cannot be performed [11]. In this short communication, we share our experiences and the results of PET-guided biopsies of UBU in [^18^F]PSMA-1007 PET/CT.

## Materials and methods

### Patients

Ten patients were referred to our clinic between December 2019 and December 2020 for PET/CT-guided biopsy after having a [18F]-PSMA-1007 PET/CT that showed at least one equivocal focal tracer uptake in the skeleton. Nine patients were referred for biochemically recurrent PC (mean PSA 1.83 ng/ml, range 0.32–5.18 ng/ml) and one patient for primary staging of newly diagnosed prostate cancer (PSA 110 ng/ml). Further details are as outlined in Table 1. All patients gave informed consent before intervention. Patient data were reviewed retrospectively in accordance with the regulations of the local ethics committee.

**Table 1:** Patient characteristics (*TNM*, tumour stage; *GSC*, Gleason score; PSA at time of PET/CT; *ADT*, androgen deprivation therapy) and results of biopsies

Patient	Biopsy no	Age (years)	TNM	GSC	PSA at PET (ng/ml)	Previous treatments	Biopsy location	SUVmax	Biopsy result
a	1	65	pT3b pN0 cM0 R1	4 + 5	1.28	Surgery, radiotherapy, ADT	Right ilium	5.9	Normal bone
b	2	53	pT2c pN0 cM0 R1	3 + 4	1.80	Surgery	Left pubic ramus	21.1	Normal bone
c	3	68	pT3a pN0 cM0 R0	4 + 4	0.47	Surgery	T7	47.0	Prostate cancer
d	4	66	cT3b cN0 cM0	4 + 3	110	No-primary staging	Right femur	18.2	Normal bone
d	5		cT3b cN0 cM0			No-primary diagnostic	Left ilium	10.6	Normal bone
e	6	72	pT2 cN0 cM0 R0	3 + 4	2.17	Surgery	Right ilium	11.1	Normal bone
f	7	77	pT2c pN0 cM0 R1	3 + 4	0.96	Surgery	L3	10.3	Normal bone
g	8	65	pT2c pN0 cM0 R1	4 + 3	5.18	Surgery	Left ischial tuberosity	5.1	Normal bone
h	9	79	pT2c pN0 cM0 R1	3 + 4	0.68	Surgery	Right pubic ramus	26.0	Normal bone
i	10	74	cT2 cN0 cM0	3 + 4	3.63	Brachytherapy	Left ilium	7.4	Normal bone
j	11	51	pT2c pN0 L0 R0	3 + 4	0.32	Surgery	T12	9.4	Normal bone

### Imaging procedures and evaluation

Imaging was performed at 90 min postinjection (p.i.) of [18F]PSMA-1007 (mean 240 MBq, range 213–254 MBq) using either an analogue (Siemens mCT, patients: a, e, f) or a digital PET system (Siemens Vision 600 (patients: b, c, d, g, h, i) or ultra-long field of view Siemens Vision Quadra (patient: j). Reconstruction parameters and imaging protocols are as previously published [7, 12]. Images were reviewed by a dual-board-certified radiologist/nuclear medicine physician (first author) and a board-certified nuclear medicine physician (last author) in consensus with image analyses performed using appropriate software (Siemens syngo.via). The foci were classified in consensus as to either high risk of malignancy, equivocal or low risk of malignancy, and uptake was measured using SUVmean and SUVmax as previously published [7]. Scrutiny of clinical notes was performed for subsequent clinical follow-up data that could confirm or refute benign or malignant biopsy findings according to a composite standard of truth (CSOT) as previously published [6]. [18F]PSMA-1007 was obtained from a radiopharmaceutical company and was produced according to good manufacturing practice (GMP) standards and conformed to national radiopharmaceutical quality standards.

### PET-guided biopsies

All patients were referred for PET-guided biopsy at the request of multidisciplinary boards for the workup of UBU found at [^18^F]PSMA-1007 PET/CT. If more than one focus was classified as potentially suspicious, the biopsy location was determined according to SUVmax of the focus as well as safe accessibility with regard to critical structures (e.g. nerves, blood vessels, etc.). In one patient (patient d), biopsies of two suspicious foci were requested. Biopsies were performed as previously described [11, 13] with verification of needle position prior to biopsy. Further details are as outlined in the Supplementary materials. All biopsies were performed by an experienced dual board-certified nuclear medicine physician and radiologist (first author).

### Histology

All material was examined on haematoxylin and eosin (H&E)-stained sections for the presence or absence of PC metastases and further histopathological findings. Additional PSMA immunohistochemical (IHC) staining was performed with details outlined in Supplementary materials. A board-certified pathologist (third author) experienced in urogenital pathology performed interpretation of H&E and PSMA-stained slides.

### Statistical analysis

For this small cohort of patients, descriptive statistics were used to compare differences in radiotracer uptake (median ± range). The small number of data points precluded any statistical significance testing.

## Results

The outcomes of each biopsy are as detailed in Table 1. A total of 11 UBU in 10 patients were biopsied. None of the UBU presented with a morphological correlate in the CT scan. All biopsies were clinically well tolerated.

**Fig. 1:**
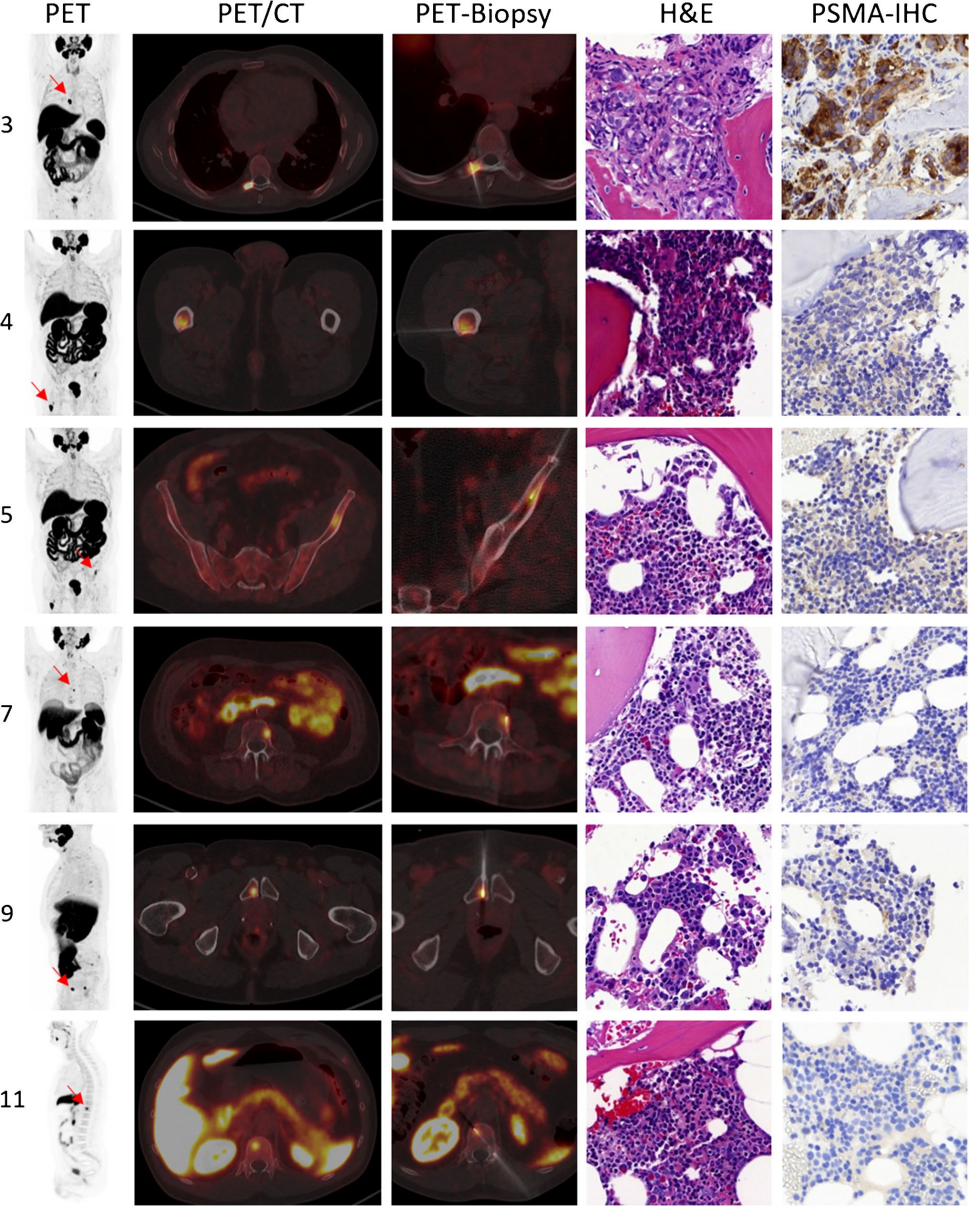
Shown are 6 example biopsies with biopsy number (c.f. Table 1), maximal intensity projection (MIP) and PET/CT, example biopsy images showing verification of needle placement and results of the histopathological analysis, which was positive for only one patient (biopsy no. 3)

In summary, 1/11 (9.1%, 95% confidence interval (CI) 1.6–37.7%) of the foci biopsied was confirmed as a bone metastasis of PC with intense PSMA expression at IHC, while 10/11 (90.9%, 95% CI 62.2–98.4%) foci were revealed to be unremarkable bone tissue without evidence of PSMA expression at IHC. Example foci shown in the PET/CT and the histopathology results are presented in Fig. 1. Histopathology for the nonmalignant UBU was unremarkable, with no indication of a secondary bone pathology or metastasis of a second malignancy.

All foci with a radiotracer uptake (local relapse, lymph nodes, bone foci, etc.) were assessed as being at high risk of malignancy, equivocal or low risk of malignancy by two experienced readers in consensus as described above. Amongst all foci examined, those judged to be at high risk of malignancy (*n* = 17) had higher uptake of [^18^F]PSMA-1007 than those judged to be equivocal (*n* = 20) or low risk (*n* = 20): SUVmean high risk: 12.2 ± 7.4, equivocal: 4.3 ± 1.6, low risk: 3.5 ± 1.3; SUVmax high risk: 18.9 ± 12.1, equivocal: 6.7 ± 2.1, low risk: 7.7 ± 10.6.

**Fig. 2:**
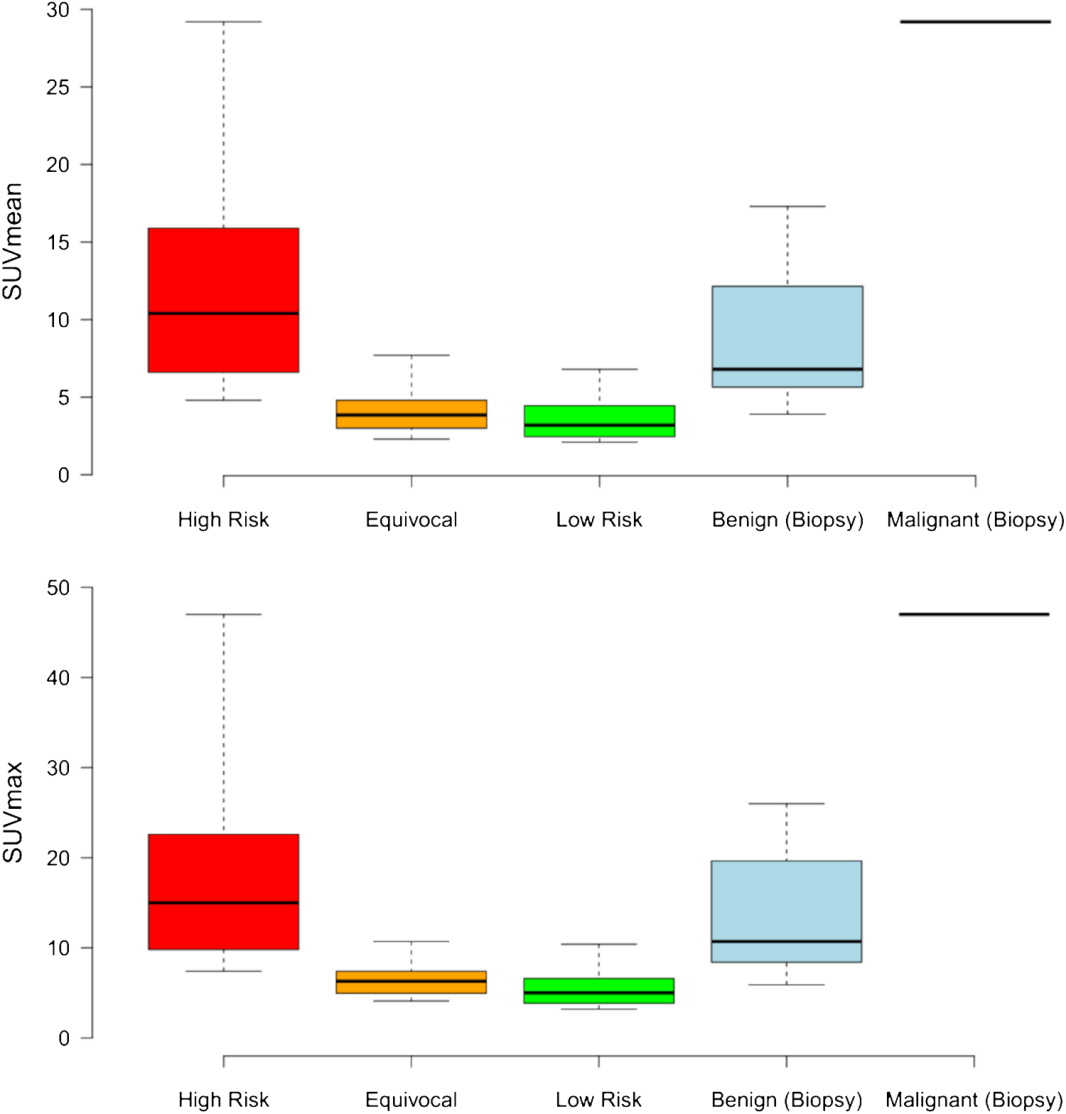
Shown are the SUVmean (above) and SUVmax (below) for foci judged clinically to be at high risk (red, *n* = 17), equivocal (orange, *n* = 20), or low risk (green, *n* = 20), as well as those foci biopsied and demonstrated to be benign (blue, *n* = 10) or the single PC lesion (black line, right)

Amongst those bone foci, which were assessed by biopsy, eight were visually classified as being at high risk of malignancy in the PET/CT (SUVmean 12.0 ± 8.1; SUVmax 18.8 ± 13.1), three as equivocal (SUVmean 4.6 ± 2.1; SUVmax 7.2 ± 3.0), and zero as low risk. None of the bone foci presented with a CT correlate. The single focus demonstrated to be a PC metastasis showed intensive uptake (SUVmean 29.2, SUVmax 47). However, as can readily be appreciated from Fig. 2, substantial overlap in uptake values occurred between foci demonstrated to be benign in origin and those judged to be at high risk of malignancy. Clinical follow-up was performed for all patients. Six patients were lost to follow-up despite all reasonable efforts to contact the patients’ physicians. For the four patients where follow-up was available, no conclusive standard of truth was available, for example for three patients despite clear fall in PSA post-stereotactic radiotherapy with the UBU outside the radiation volume, the PSA remained measurable.

## Discussion

We present the first published cohort of PET-guided biopsies for confirmation or refutation of PC in UBU for [^18^F]PSMA-1007 in a series of 10 patients. We found that only one out of eleven foci biopsied was a metastasis of PC. Similar results were reported in a series of bone foci with follow-up, albeit in a small cohort of patients (*n* = 15) with primary PC [10]. Nevertheless, we interpret these studies with small cohorts with caution; larger and better-powered studies are required to estimate the true scale of the problem. Moreover, the number of reports of invasive measures to clarify UBU, including rib resection [14, 15], suggest that occasionally, diagnostic dilemmas can occur, which warrant highly invasive measures and are a clinically relevant issue. Although SUV cutoff values have been advanced as a means of differentiating between benign and malignant foci [4], we find in our series of biopsies a substantial overlap between foci judged to be clinically at high risk and with high tracer avidity, yet with benign histology. Moreover, Grünig et al. find that even with follow-up, 43% of UBU remain unclear, further demonstrating that there is currently no satisfactory method to differentiate reliably between UBU in [^18^F]PSMA-1007 PET/CT [5]. In contrast to the more established [^68^ Ga]Ga-PSMA-11, where prospective diagnostic accuracy studies have been performed [16], only limited data are available for [^18^F]PSMA-1007 with only preliminary observations in small cohorts [17], in mixed cohorts of primary and recurrent PC, [18] thereby with limited interpretability, or without any verification of positive findings [19, 20]. Further studies are urgently required to determine the true diagnostic accuracy of [^18^F]PSMA-1007, especially for bone foci.

Interestingly, despite intensive avidity for [^18^F]PSMA-1007 and that a substantial proportion of the foci biopsied were judged clinically to be at high risk of malignancy, no evidence of PSMA expression could be demonstrated at IHC. Previous reports of nonspecific salivary gland uptake posit a non-PSMA-related uptake mechanism [21]. The lack of IHC PSMA expression, and lack of histopathological evidence for benign, inflammatory, or other malignant entities, suggests a potential non-PSMA-receptor-mediated uptake mechanism for UBU in [^18^F]PSMA-1007 PET/CT; further studies are needed to elucidate the true mechanism for this, which remains unclear. For example, variation in molar activity has been demonstrated to influence the biodistribution of some PSMA-radioligands [22]. Quality control of the GMP manufactured radiopharmaceutical and the high frequency with which UBU are observed in other centres makes a local radiochemical purity problem unlikely [4, 5].

There was a lack of histopathological evidence indicative of PC or other inflammatory or neoplastic processes suggestive of a non-PC malignancy. Although PSMA-negative PC can occur in a minority of patients [23], this cannot explain the discordance between PSMA PET/CT and IHC findings and points towards an as yet unclarified issue with the radiopharmaceutical. Noting the abundance of reports of UBU and being cognisant of their potential adverse clinical impact, further studies are necessary to understand better this phenomenon and the limitations of this tracer. Meanwhile, we find PET biopsy to be a useful and minimally invasive method of clarifying UBU, particularly where, in the absence of a CT correlate, traditional CT-guided biopsy is not possible and where clinical interpretation of imaging findings or SUV cannot reliably differentiate between benign and malignant UBU. All biopsies could either confirm or refute clinically suspicious foci, enabling more accurate staging.

This short communication with clinical data is primarily limited by its small cohort size as well as the necessarily undefined selection criteria for patients referred for a biopsy on clinical grounds. A further weakness was the lack of useful data in the follow-up period, which could clarify the non-biopsied UBU. Future systematic studies, including biopsies of low-risk foci, are warranted to evaluate better the true scale of this problem and to evaluate what the true incidence of malignancy is amongst UBU in [^18^F]PSMA-1007 PET/CT.

## Conclusion

UBU are common in [^18^F]PSMA-1007 PET/CT. In our small cohort, one of eleven biopsies represented a true metastasis. For those confirmed as benign, no IHC evidence of PSMA expression was found, suggesting a non-PSMA-receptor-mediated mechanism for focal bone uptake, the cause of which remains unclear. Readers must interpret focal bone uptakes of [^18^F]PSMA-1007 with caution in order to reduce the risk of erroneous staging and treatment of patients, particularly where our cohort suggests that reliance on interpretation of SUV or clinical characteristics does not reliably differentiate between benign and malignant foci. We endorse the use of PET-guided biopsy as a useful and minimally invasive option to clarify focal bone uptakes of [^18^F]PSMA-1007, particularly where the lack of CT correlate makes CT-guided biopsy impossible and where the subsequent treatment is critically dependent on the characterization of the bone lesion.

## Supplementary Information

Below is the link to the electronic supplementary material.

Supplementary file1 (DOCX 13 KB)
