# Supplementary material for: Assessment of malignancy and PSMA expression of uncertain bone foci in [18F]PSMA-1007 PET/CT for prostate cancer—a single-centre experience of PET-guided biopsies
Source: Eur J Nucl Med Mol Imaging. 2022 Apr 28;49(11):3910–6. doi: 10.1007/s00259-022-05745-5 (PMC9399054; doi:10.1007/s00259-022-05745-5)
Supplement: Supplementary file 1 — Supplementary file1 (DOCX 13 KB) [file 259_2022_5745_MOESM1_ESM.docx]

**Supplementary Materials**

*Biopsy Procedure*

Patients were positioned in prone or supine position depending on the location of the suspected lesion and a PET/CT (mean dose 191 MBq [^18^F]PSMA-1007, range 97 – 249 MBq) over the region of interest was performed for biopsy planning. If necessary acquisition time was prolonged to assure optimal image quality (max. 5 min. / bed position). Interventions were done under aseptic conditions, local anaesthesia and, if necessary, under anxiolysis with lorazepam *per os*. Depending on the location of the lesion of interest, either a 15 G or a 12 G coaxial bone biopsy system was chosen. A step-wise, CT-guided introduction of the biopsy needle was performed. An additional PET/CT-scan immediately prior to sampling was performed to assure correct needle position. One or two samples out of each lesion were obtained and directly embedded in formalin for further histopathological and immunohistochemical work up.

*Histology Procedure*

Additional immunohistochemical staining was performed on 4μm sections by automated staining using the Bond RX (Leica Biosystems). Tris buffer at 95° for 30 min was used for antigen retrieval (Leica Biosystems). The PSMA antibody clone M3620 (Dako-Agilent) was diluted 1:400 and incubated by room temperature for 30 min. Visualization was performed with the Bond Polymer Refine Detection Kit (Leica Biosystems) according to the manufacturer guidelines.
